# Is clinical primary care surveillance for tularaemia a useful addition to laboratory surveillance? An analysis of notification data for Finland, 2013 to 2019

**DOI:** 10.2807/1560-7917.ES.2022.27.4.2100098

**Published:** 2022-01-27

**Authors:** Charlotte C Hammer, Timothee Dub, Oskari Luomala, Jussi Sane

**Affiliations:** 1 European Programme for Intervention Epidemiology Training (EPIET), European Centre for Disease Prevention and Control (ECDC), Stockholm, Sweden; 2 Department of Health Security, Finnish Institute for Health and Welfare (THL), Helsinki, Finland

**Keywords:** surveillance, tularaemia, clinical primary care surveillance, vector-borne diseases

## Abstract

**Background:**

In Finland, surveillance of tularaemia relies on laboratory-confirmed case notifications to the National infectious Diseases Register (NIDR).

**Aim:**

The aim of the study was to assess the suitability and usefulness of clinical surveillance as an addition to laboratory notification to improve tularaemia surveillance in Finland.

**Methods:**

We retrieved NIDR tularaemia surveillance and primary healthcare data on clinically diagnosed tularaemia cases in Finland between 2013 and 2019. We compared incidences, demographic distributions and seasonal trends between the two data sources.

**Results:**

The median annual incidence was 0.6 (range: 0.1–12.7) and 0.8 (range: 0.6–7.2) per 100,000 for NIDR notifications and primary healthcare notifications, respectively. Cases reported to NIDR were slightly older than cases reported to primary healthcare (median: 53 years vs 50 years, p = 0.04), but had similar sex distribution. Seasonal peaks differed between systems, both in magnitude and in timing. On average, primary healthcare notifications peaked 3 weeks before NIDR. However, peaks in NIDR were more pronounced, for example in 2017, monthly incidence per 100,000 of NIDR notifications peaked at 12.7 cases in September, while primary healthcare notifications peaked at 7.2 (1.8 ratio) in August.

**Conclusions:**

Clinically diagnosed cases provide a valuable additional data source for surveillance of tularaemia in Finland. A primary healthcare-based system would allow for earlier detection of increasing incidences and thereby for early warning of outbreaks. This is crucial in order to implement targeted control and prevention measures as early as possible.

## Introduction

Tularaemia is a bacterial zoonosis caused by *Francisella tularensis* [1-3]. Human infections, particularly with the *F. tularensis* subspecies *tularensi*s are potentially severe, with a case fatality rate (CFR) of up to 15% without antibiotic treatment and a CFR of 2% with antibiotic treatment [2]. However, the subspecies found in Finland, *F. tularensis* *holartica* (type B), is considered less virulent and has a less severe clinical presentation [3]. There are four main routes of transmission: ingestion of contaminated food or water, direct contact with infected animals, inhalation of contaminated dust and vector-borne transmission [2-4]. The latter is the main route of transmission in Finland and Sweden. It is associated with *Aedes cinereus* mosquitoes [3] with a seasonal peak in late summer to autumn [4]. Hence, large mosquito populations are an established risk factor for tularaemia outbreaks in these countries [5]. Ecologically, the main foci of tularaemia are boreal forest taiga, temperate broadleaf and mixed forest, and temperate grassland and shrubland [2].

Tularaemia is found in most of the northern hemisphere and in Europe [2]. The expected reported number of cases in Europe is ca 800 per year [2]. The highest incidence of tularaemia in Europe is reported in endemic areas in Finland and Sweden [6] and both countries see recurring outbreaks of tularaemia [7-9]. The disease seems to follow a cyclical pattern in the Nordic region with peaks every 2–3 years, probably associated with peaks in the vole population during the previous year [7]. In 2000, 2% of the Finnish population showed a serological response to *F. tularensis* [7].

Currently, the main tularaemia surveillance system in Finland is the National Infectious Diseases Register (NIDR) which consists of laboratory notifications. However, clinical surveillance – in Finland this consists of notification data from the primary healthcare system (Avohilmo) – has the potential to be a more timely source for outbreak detection and may complement laboratory-based surveillance. This approach has been studied and validated for the surveillance of Lyme borreliosis with the conclusion that clinical surveillance was a suitable and useful addition to laboratory-based surveillance of that disease [10].

We here describe the epidemiology of tularaemia in Finland between 2013 and 2019 using data from the current laboratory-based national surveillance system (NIDR) and from the primary healthcare notifications system (Avohilmo) to assess the suitability of primary healthcare notifications for tularaemia outbreak detection and guidance of prevention and control measures.

## Methods

### Study population

The entire population of Finland (5.5 million inhabitants) was eligible for inclusion in the study. This means that all 20 geographically and administratively defined healthcare districts and the Åland Islands were included. Populations of these districts ranged from 63,000 to 1.8 million in 2019.

### Study design

We performed a retrospective analysis of registry data from the Avohilmo and NIDR systems. We analysed demographic characteristics of cases in both systems, spatiotemporal characteristics and the specifics of the 2016 outbreak year to further assess the timeliness of Avohilmo compared with NIDR.

### Data sources

#### National Infectious Diseases Register for microbiologically confirmed tularaemia cases

Routine surveillance of tularaemia in Finland is laboratory-based. Microbiological laboratories performing tularaemia diagnostics in Finland notify the NIDR electronically of any positive findings. Each notification includes the following information: date of specimen collection, each patient’s unique national identity code, date of birth, sex and place of residence. For this study, we extracted all microbiologically confirmed tularaemia cases from NIDR that were reported during the period 2013 to 2019. For analysis we used the date of specimen collection. The most common type of laboratory confirmation during our study period was serology (96%).

#### Avohilmo for primary healthcare visits

Since 2011, outpatient healthcare visits with the International Classification of Diseases, 10th Revision (ICD-10) code for A21 ('tularaemia') at primary healthcare units (municipal health centres and health centre wards) have been registered. Notifications in Avohilmo include the patient’s national identity code, age, sex, place of healthcare service, information concerning healthcare admission, investigations, treatment and discharge diagnoses according to the ICD-10. Tularaemia cases in Avohilmo were defined as clinical cases, regardless of microbiological confirmation reported with the ICD-10 code A21. Information about clinical manifestation or subclassification was not available. We used Avohilmo data to estimate the number of clinically diagnosed tularaemia cases in Finland during the period from 2013 to 2019. For analysis we used the date of the first healthcare visit associated with the A21 ICD-10 code. In Finland, clinical diagnosis can be done based on symptoms alone for the purpose of starting treatment early; it is per the current guidelines supposed to be confirmed by antibody testing or in some circumstance by culture from pus or PCR [11].

### Data analysis

Case numbers and incidences of tularaemia in Finland were calculated and compared for the two systems. In addition, analysis by healthcare district was done for the year 2016, when a major outbreak took place. We compared data from NIDR and Avohilmo and analysed the delay in the peak of the 2016 outbreak in NIDR compared with Avohilmo. Demographic characteristics were analysed using t-test (age) and chi-squared test (sex). Analysis was done in R (version 3.6.1 [12] using RStudio) and MS Excel with additional mapping done in QGIS (version 3.4).

### Ethical statement

The research did not require ethical review before implementation as its aim was related to analysis of routine surveillance data as part of the core statutory missions of the Finnish Institute for Health and Welfare.

## Results

The total number of cases between January 2013 and December 2019 was 712 in Avohilmo and 915 in NIDR.

### Demographic characteristics of tularaemia cases

Demographic characteristics were similar in NIDR and Avohilmo with 53% of male cases in both systems. Cases registered in Avohilmo were slightly younger, with a median of 50 years (interquartile range (IQR): 33-68) compared with 53 years (IQR: 40-67) in NIDR (p = 0.04).

### Analysis of tularaemia cases and incidence

**Figure 1:**
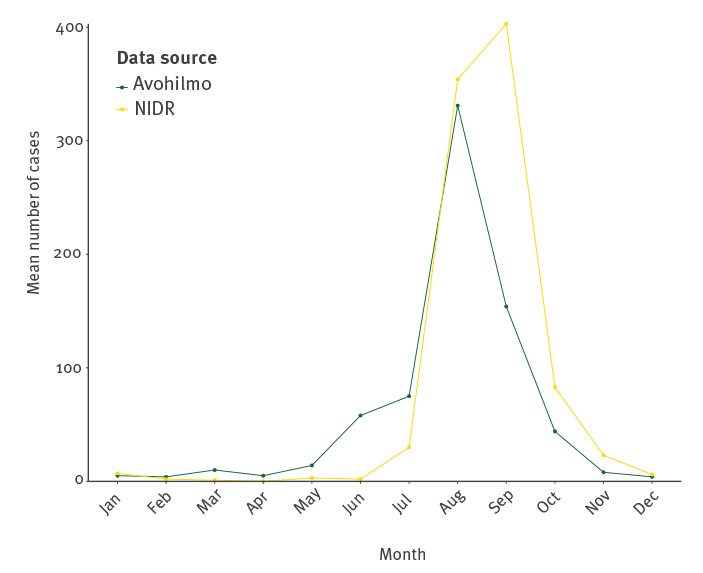
Monthly mean tularaemia cases, Finland, 2013–2019 (NIDR n = 915; Avohilmo n = 712)

**Figure 2:**
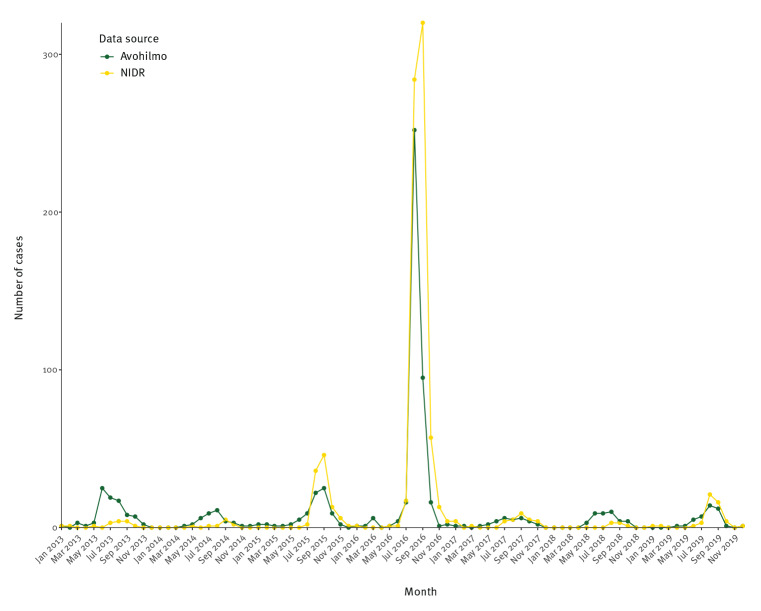
Monthly tularaemia incidence, Finland, January 2013–December 2019 (NIDR n = 915; Avohilmo n = 712)

A seasonal pattern could be observed throughout the years. Annual peak time was August in Avohilmo and September in NIDR (Figures 1 and 2). Between 2013 and 2019, the mean annual incidence was 2.4 NIDR notifications per 100,000 (median: 0.6), ranging from 0.1 to 12.7, and 1.9 primary healthcare notifications per 100,000 (median: 0.8), ranging between 0.6 and 7.2. The raw data for each district are provided in Supplementary Tables S1 and S2.

NIDR: National infectious Diseases Register.

NIDR: National infectious Diseases Register.

### Analysis by geographical distribution

**Figure 3:**
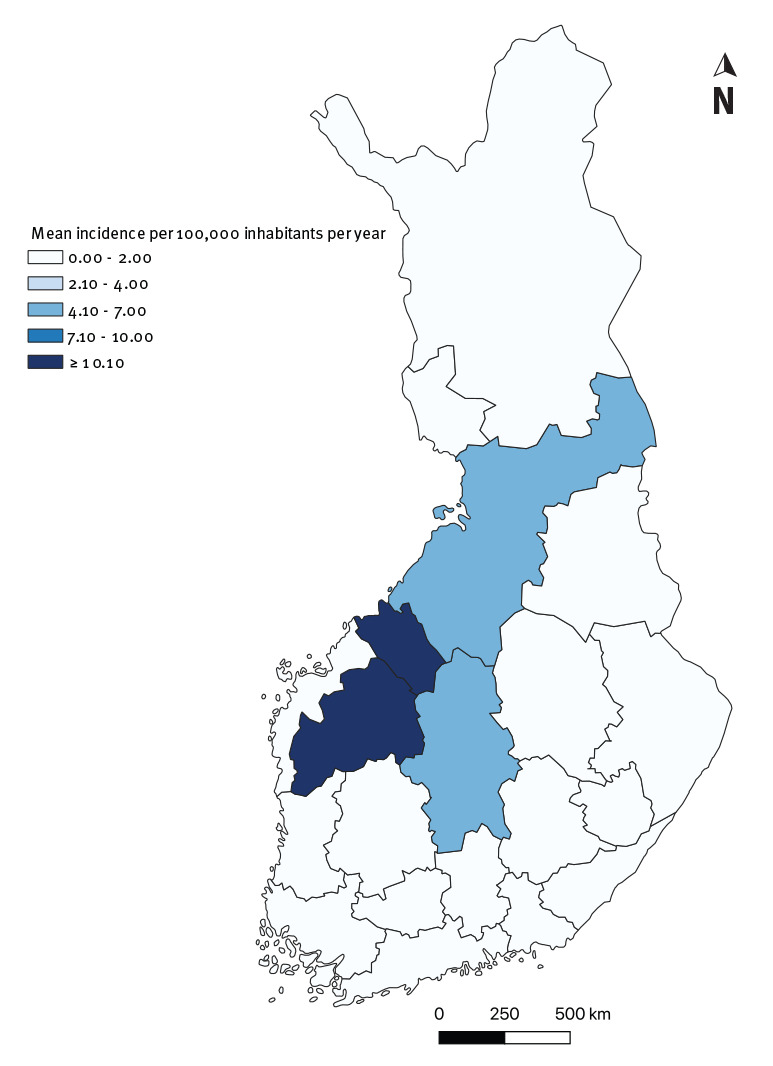
Mean incidence of clinically diagnosed tularaemia, by region, Finland, 2013–2019 (n = 712)

**Figure 4:**
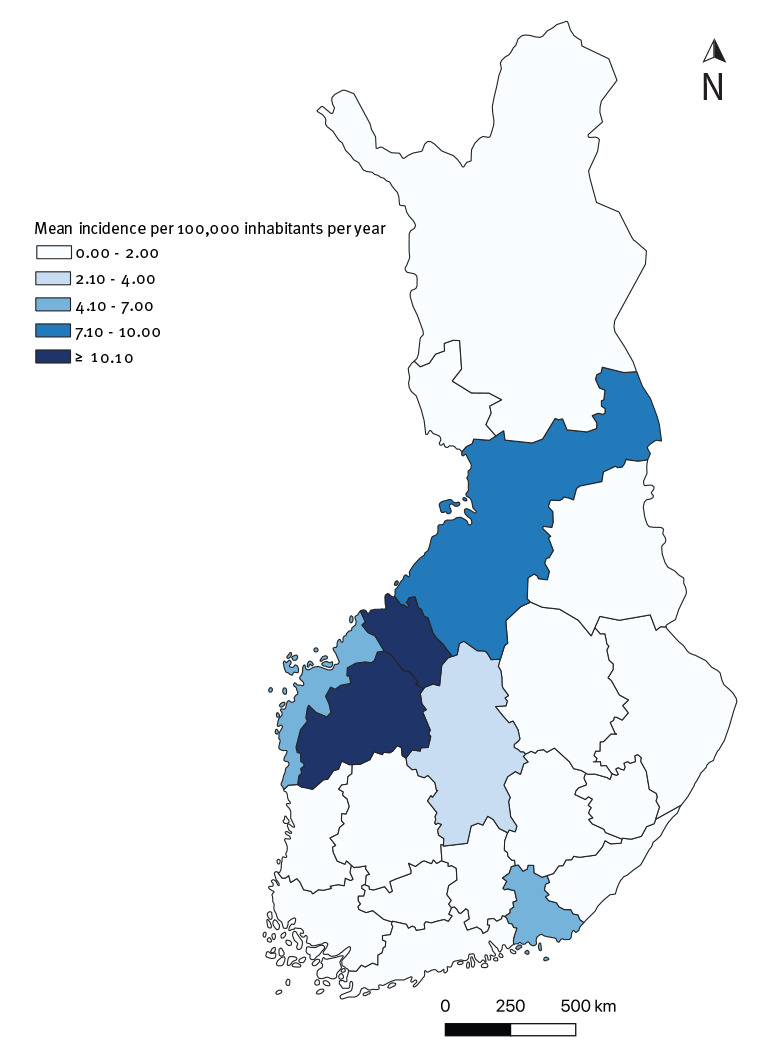
Mean incidence of laboratory-confirmed tularaemia, by region, Finland, 2013–2019 (n = 915)

The geographical distribution of cases was similar in NIDR and Avohilmo (Figures 3 and 4). The Ostrobothnia region in western Finland was the main geographical focus of tularaemia in Finland (Figures 3 and 4). It had the highest incidences per 100,000 inhabitants throughout the 7-year period and was at the centre of the 2016 outbreak.

### The 2016 outbreak

**Figure 5:**
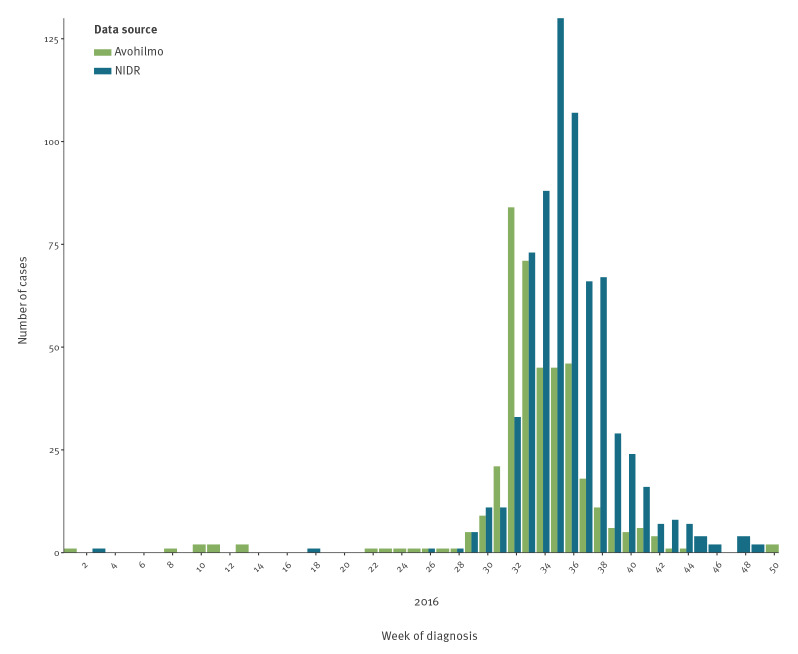
Tularaemia cases by week of diagnosis, Finland, January 2016–December 2016 (NIDR n = 915; Avohilmo n = 712)

A large outbreak of tularaemia in 2016 was identifiable in both Avohilmo and NIDR (Figure 5). In NIDR, 698 cases were recorded during that year. In Avohilmo, 395 cases were recorded during the same period. Annual incidences in both systems peaked at 12.7 cases per 100,000 in NIDR and at 7.2 per 100,000 in Avohilmo. Based on the weeks with the highest case counts, NIDR had a 3-week delay compared with Avohilmo, with the peak occurring in week 31 in Avohilmo and in week 34 in NIDR. 

NIDR: National infectious Diseases Register.

The outbreak occurred in Western Finland, predominantly in the Ostrobothnia region. This region consists of three healthcare districts: Northern Ostrobothnia, Central Ostrobothnia and Southern Ostrobothnia, which are generally the districts with the highest annual incidences in Finland. During the 2016 outbreak year, incidence in these three districts peaked at, respectively, 16.8, 108.4 and 64.1 per 100,000 in Avohilmo. In the same year, incidences peaked at, respectively, 41.1, 118.9 and 96.7 per 100,000 in NIDR. Outside of the outbreak year, the incidence in these three districts ranged from 0.0 (Central Ostrobothnia) to 6.6 per 100,000 (Central Ostrobothnia) in Avohilmo and from 0.0 (Central and Northern Ostrobothnia) to 15.4 per 100,000 (Northern Ostrobothnia) in NIDR.

## Discussion

This study has shown that the Avohilmo system is suitable for tularaemia surveillance and is more sensitive to increases in case numbers than the currently used NIDR system. Between 2013 and 2019, the median annual incidence was 0.6 NIDR notifications per 100,000 and 0.8 primary healthcare notifications per 100,000. Age and sex distribution were similar across the two systems. Both systems showed the expected seasonal pattern of tularaemia in Finland. Peak months were August for Avohilmo and September for NIDR across the 7-year period.

Overall, while Avohilmo does not always show peaks of the same magnitude as NIDR, it usually, even though not every season, shows them earlier. In the years when the peak was not earlier, it was in the same month in Avohilmo and in NIDR but cases increased earlier towards the peak in Avohilmo. One potential reason why Avohilmo has a smaller reporting fraction and hence does not show the same magnitude of peaks might be because, unlike NIDR, it only covers patients accessing the public healthcare system in Finland, whereas NIDR covers all laboratory diagnoses, irrespective of whether the patient has been examined in public, private or occupational healthcare. However, when assessing the utility of clinical surveillance for tularaemia, more important than the magnitude is the sensitivity to increases in case numbers and therefore the possibility of earlier outbreak detection. Looking at the 2016 outbreak, the delay for the peak in NIDR was ca 3 weeks. This is a considerable time period if such data are used to guide outbreak response and preparedness activities based on outbreak thresholds. This demonstrates that, just like syndromic surveillance, this kind of clinical surveillance has the potential to increase timeliness of signal detection, which can be considered its main benefit [13-15].

Early outbreak detection is key for public health interventions. If the surveillance system allows for near real-time outbreak detection, it can act as an early warning system and guide targeted risk communication [16,17]. These could include information on risky behaviours and areas as well as information on how to recognise a tularaemia infection. In addition, considering the mainly vector-borne nature of tularaemia in Finland, early identification of hotspots could also lead to targeted vector control interventions if applicable. Thus, ideally, tularaemia outbreaks could be controlled quicker and with fewer cases overall, leading to reduced morbidity and work absenteeism. Using a variety of data sources, including clinical surveillance based on the Avohilmo system, allows for more timely and more targeted outbreak detection and intervention, potentially reaching near-real time. The obvious downside to this increased timeliness is a higher rate of misclassification in such a surveillance system compared with one that relies on laboratory confirmation [18,19].

This study was subject to certain biases and limitations. We used the date of specimen collection for NIDR and the date of the first healthcare visit for Avohilmo. However, we are aware that there is a time delay between onset of symptoms and laboratory confirmation and that this is potentially larger than the time delay between onset of symptoms and visiting a healthcare professional. We did specifically not perform a register linkage study in this case, which would require significant further work as well as different ethical requirements. This lack of a linkage study prevents having information about the average time of delays or the overlap between the two systems.

In addition, actual case numbers may have been underestimated in both systems. No established correction factor exists for under-reporting of tularaemia cases based on treatment without laboratory diagnosis. However, the impact of milder cases not seeking healthcare can be estimated to be small and most cases can be assumed to present with distinct acute clinical symptoms [7,16]. Finally, regions that are more familiar with tularaemia may be more likely to correctly identify tularaemia cases. For Avohilmo, this could have led to different levels of clinical awareness of tularaemia across regions. In addition, this might have impacted the likelihood of seeking laboratory confirmation in different regions.

## Conclusion

Clinical surveillance for tularaemia in Finland through the Avohilmo system is both consistent with NIDR and detects outbreaks earlier. This suggests that Avohilmo is a good data source for early outbreak detection and could inform timely public health recommendation including targeted risk communication. We recommend the use of the system for this purpose as a suitable addition to laboratory-based surveillance and suggest building on the work presented in this paper by developing a formal mechanism for the development of outbreak thresholds in Avohilmo to be used as an early warning system. In light of the reoccurring nature of larger tularaemia outbreaks in Finland it is desirable to establish outbreak thresholds and targeted risk communication and prevention. Future studies should include both investigations regarding outbreak thresholds as well as register linkage between Avohilmo and NIDR to understand how much these two systems overlap and establish if clinical diagnosis alone is happening in certain cases or if all clinically diagnosed cases are also later laboratory-confirmed and hence reported in NIDR. More generally, our findings suggest that clinical surveillance can be a suitable addition to laboratory surveillance for tularaemia. This would be even more useful if clinical manifestations were recorded, which is currently not the case in the Finnish system.

## Supplementary Data

113000 Supplement
