# Supplementary material for: Is clinical primary care surveillance for tularaemia a useful addition to laboratory surveillance? An analysis of notification data for Finland, 2013 to 2019
Source: Euro Surveill. 2022 Jan 27;27(4):2100098. doi: 10.2807/1560-7917.ES.2022.27.4.2100098 (PMC8796291; doi:10.2807/1560-7917.ES.2022.27.4.2100098)
Supplement: Supplement [file 21-00098_HAMMER_Supplement.pdf]

Supplementary Material

This supplementary material is hosted by *Eurosurveillance* as supporting information alongside the article ‘Is clinical primary care surveillance for tularaemia a useful addition to laboratory surveillance? An analysis of notification data for Finland, 2013 to 2019’ on behalf of the authors who remain responsible for the accuracy and appropriateness of the content. The same standards for ethics, copyright, attributions and permissions as for the article apply. Supplements are not edited by Eurosurveillance and the journal is not responsible for the maintenance of any links or email addresses provided therein."

Supplementary Table S1: Incidence of laboratory confirmed tularemia in Finland (per 100,000 population) by district, 2013-2019.

Healthcare District	2013	2014	2015	2016	2017	2018	2019
**Ahvenanmaa**	0.00	0.00	0.00	0.00	0.00	0.00	0.00
**Etelä-Karjala**	0.00	0.00	0.00	1.60	0.00	0.00	0.00
**Etelä-Pohjanma**	2.01	1.01	6.59	96.66	3.58	0.51	2.59
**Etelä-Savo**	0.00	0.00	0.00	1.03	0.00	0.00	0.00
**HUS**	0.13	0.06	0.43	2.93	0.18	0.00	0.06
**Itä-Savo**	0.00	0.00	0.00	0.00	0.00	0.00	0.00
**Kainu**	0.00	0.00	0.00	0.00	0.00	0.00	1.38
**Kanta-Häme**	0.00	0.00	0.00	0.58	0.00	0.00	0.00
**Keski-Pohjanma**	2.65	0.00	11.89	118.97	3.84	0.00	2.59
**Keski-Suomi**	1.45	0.36	0.73	11.59	0.79	0.79	3.96
**Kymenlaakso**	0.00	1.16	0.00	27.52	0.59	0.60	0.00
**Lapi**	0.00	0.00	0.00	4.25	0.00	0.00	0.00
**Länsi-Pohja**	0.00	0.00	1.59	4.80	1.62	0.00	0.00
**Pirkanma**	0.00	0.20	0.60	4.34	0.56	0.19	0.93
**Pohjois-Karjala**	0.00	0.00	0.00	0.00	0.00	0.00	0.00
**Pohjois-Pohjanma**	0.25	0.00	15.36	41.10	1.47	0.00	4.39
**Pohjois-Savon**	0.00	0.00	0.00	0.00	0.41	0.00	0.41
**Päijät-Häme**	0.00	0.00	0.00	0.00	0.00	0.47	0.00
**Satakunna**	0.00	0.89	0.00	9.92	0.45	0.46	0.46
**Vaasa**	0.59	0.00	1.76	32.92	1.18	0.00	0.59
**Varsinais-Suomi**	0.21	0.21	0.63	2.09	0.42	0.00	0.62
**All of Finland**	0.28	0.18	1.90	12.68	0.58	0.13	0.87

Supplementary Table S2: Incidence of clinically diagnosed tularemia in Finland (per 100,000 population) by district, 2013-2019.

Healthcare District	Incidence 2013	Incidence 2014	Incidence 2015	Incidence 2016	Incidence 2017	Incidence 2018	Incidence 2019
Ahvenanmaa	0.00	0.00	0.00	0.00	0.00	0.00	0.00
Etelä-Karjala	1.58	1.58	0.79	0.00	0.00	2.33	0.00
Etelä-Pohjanma	6.04	2.02	2.53	64.10	4.09	4.12	2.07
Etelä-Savo	1.01	1.01	0.00	0.00	0.00	0.00	0.00
HUS	1.14	0.37	0.56	0.92	0.06	0.18	0.24
Itä-Savo	3.63	0.00	0.00	0.00	2.37	0.00	0.00
Kainu	1.30	0.00	0.00	1.34	0.00	1.37	0.00
Kanta-Häme	0.00	0.57	0.57	0.58	0.00	0.00	0.00
Keski-Pohjanma	2.65	0.00	6.60	108.40	6.40	1.29	2.59
Keski-Suomi	4.00	1.09	2.54	13.76	1.19	2.77	3.96
Kymenlaakso	0.58	0.00	0.58	5.86	2.37	0.60	0.00
Lapi	0.85	0.00	0.00	0.85	0.85	0.00	0.00
Länsi-Pohja	0.00	1.57	0.00	0.00	0.00	0.00	0.00
Pirkanma	0.80	0.60	0.40	2.17	0.56	0.37	0.74
Pohjois-Karjala	1.77	1.18	1.78	0.00	0.00	0.00	0.00
Pohjois-Pohjanma	1.48	0.98	5.37	16.78	0.24	1.22	2.93
Pohjois-Savo	2.01	1.21	2.42	2.02	0.00	1.63	0.82
Päijät-Häme	2.36	1.90	0.48	0.00	0.47	0.00	0.00
Satakunna	2.67	0.45	0.90	1.35	0.45	0.46	0.00
Vaasa	0.00	0.00	1.76	9.99	0.59	0.00	0.00
Varsinais-Suomi	0.42	0.42	0.84	0.84	0.21	0.42	0.41
All of Finland	1.58	0.69	1.46	7.18	0.58	0.71	0.76
